# Vitamin D supplementation in the treatment of type 2 diabetic microangiopathy: A protocol for a systematic review and meta-analysis

**DOI:** 10.1097/MD.0000000000020978

**Published:** 2020-08-14

**Authors:** Junmin Chen, Xiayu Gong, Jie Liu, Tingting Wang, Xiaoyan Shi, Xiaoran Zhang, Qiu Chen

**Affiliations:** Hospital of Chengdu University of Traditional Chinese Medicine, Chengdu, Sichuan Province, P.R. China.

**Keywords:** meta-analysis, protocol, systematic review, type 2 diabetic microangiopathy, vitamin D

## Abstract

**Background:**

The number of people with diabetes is growing exponentially.Human studies have shown that vitamin D supplementation is beneficial for type 2 diabetic microangiopathy. However, owing to the low quality, small sample size, and methodological heterogeneity of these studies, this conclusion is not convincing. Consequently, in order to determine whether vitamin D supplementation is effective and safe in type 2 diabetic microangiopathy, it is necessary to conduct a meta-analysis of high-quality clinical trials.

**Methods:**

We will search each database from the built-in until March 2020. The English literature mainly searches Cochrane Library, PubMed, EMBASE, and Web of Science, while the Chinese literature comes from CNKI, CBM, VIP, and Wangfang database. Simultaneously we will retrieval clinical registration tests and grey literatures. In this study, only the clinical randomized controlled trials were selected to evaluate the efficacy and safety of vitamin D in the treatment of type 2 diabetic microangiopathy. The two researchers independently conducted literature selection, data extraction and quality assessment. Statistical heterogeneity among studies will be evaluated using the Cochran *Q* test (x^2^) and the I^2^ statistical value. We will utilize the Review Manage software V5.3.0 (The Nordic Cochrane Center, The Cochrane Collaboration, 2014, Copenhagen, Denmark) to statistically analyze all data.

**Ethics and dissemination:**

Ethics and dissemination: This study is a systematic review of vitamin D supplementation as a treatment of type 2 diabetic microangiopathy.

**Results:**

This study will provide high-quality synthesis of effectiveness and safety of vitamin D supplementation for type 2 diabetic microangiopathy.

**Conclusion:**

This systematic review aims to provide new options for vitamin D treatment of type 2 diabetic microangiopathy in terms of its efficacy and safety.

**Registration number:**

LNPLASY202050055

## 1 Introduction

Diabetes mellitus (DM) is a metabolic disease characterized by persistent hyperglycemia.^[1]^ Diabetes has led to a heavy medical burden and a large indirect social cost, with an annual global investment of more than $827 billion.^[2]^ In addition to maintaining calcium and phosphorus homeostasis and bone metabolism balance, vitamin D has been proved to play an important role in the occurrence and development of type 2 DM (T2DM).^[3]^ Studies have shown that vitamin D can reduce inflammation and autoimmune response, promote insulin synthesis and secretion, and play a regulatory role in the occurrence and development of diabetes and its complications.^[4]^ Vitamin D deficiency has been shown to be an important risk factor for limited secretion of islet β cells and insulin resistance.^[5]^ Based on the analysis of relevant data from 110 countries, The International Diabetes Federation predicts that the number of global diabetes patients will reach 592 million in 2035.^[6]^ Because of the difficulty of blood glucose control and poor compliance in T2DM patients, with the extension of the disease time, the complications of microvascular are often associated: diabetic nephropathy, diabetic retinopathy (DR), diabetic cardiomyopathy and so on. Undoubtedly, it is even worse in the treatment, resulting in an obvious increase in the incidence of end-stage renal disease (ESRD), cardiovascular and cerebrovascular events, blindness in both eyes and so on.^[7]^ It greatly endangers the life expectancy and quality of life of patients, but also adds a huge economic burden to the society.^[8]^ Diabetic peripheral neuropathy (DPN) is one of the most common chronic complications of DM (DM), with a high incidence rate of 90%. hyperglycemia has been considered as the main risk factor. However, blood glucose control alone can not effectively prevent the occurrence of DPN.^[9–11]^ Previous studies have found that there are nuclear receptors of vitamin D in neurons and glial cells, and vitamin D participates in the synthesis of neurotrophic factors and neurotransmitter synthetase.^[12]^ Meta analysis showed that the risk of DPN was significantly increased in type 2 diabetic patients with vitamin D deficiency (or = 2.88, *P* < .00001).^[13]^ Moreover, clinical observation and study showed that there was a significant correlation between vitamin D deficiency and neuropathic pain symptoms, neurological deficit and autonomic nervous dysfunction.^[14,15]^ DR is 1 of the main causes of moderate or severe visual impairment and blindness,^[16]^ and it is also the main cause of vision loss of working age adults in developed countries,^[17]^ which has a serious impact on the quality of life of diabetic patients. The prevalence of DR is 34.6% in the global diabetes patients, in which the prevalence of physician's desk reference is 6.96%, and the prevalence of DR that poses a threat to vision is 10.2%.^[18]^ Modjtahedi BS et al^[19]^ reported that the average number of people who are blind due to diabetes each year is up to 10000, and the risk of blindness in DM patients is significantly higher than that in non diabetic patients. The specific pathogenesis of DR remains to be further explored, but previous studies have confirmed that there is a close relationship between the course of T2DM in DR,^[20]^ and the risk of DR in T2DM patients with vitamin D deficiency is significantly higher than that in T2DM patients with normal vitamin D,and vitamin D can be an important indicator to predict the severity of DR.^[21]^ According to the data of American kidney data system, 20% to 40% of diabetic patients in the United States have kidney damage in varying degrees, and diabetic nephropathy is the first secondary factor leading to ESRD.^[22]^ Diabetic nephropathy is characterized by the appearance of microalbuminuria in the early stage. If it is not diagnosed and intervened in time, it will gradually develop into a large number of albuminuria, becoming the main cause of chronic kidney disease and ESRD, even life-threatening.^[23]^ Previous studies have shown that early diabetic kidney disease (DKD) is closely related to vitamin D deficiency,^[24]^ so it is particularly important to find a way to reverse microalbuminuria in early DKD for improving the prognosis of patients with DKD. Vitamin D has a variety of biological activities. Animal studies and clinical trials have proved that vitamin D deficiency is related to the progress of chronic kidney disease. Vitamin D supplementation or its active derivatives can improve endothelial cell damage, reduce proteinuria, reduce renal fibrosis and thus delay the progression of diabetic nephropathy. However,systematic reviews are generally more competent and less biased than the individual studies included, and the careful collection of therapeutic effects can provide the most accurate overall assessment of interventions.^[25]^ There are currently no systematic reviews to explore the therapeutic effect of vitamin D supplementation in type 2 diabetic microangiopathy. The aim of this study is to systematically evaluate the literature and meta-analyze the therapeutic effects of vitamin D supplementation in type 2 diabetic microangiopath.

## 2 Methods

### 2.1 Protocol registration

The systematic review protocol has been registered on the LNPLASY website as LNPLASY202050055. (https://inplasy.com/inplasy-2020-5-0055/). It is reported following the guidelines of Cochrane Handbook for Systematic Reviews of Interventions and the Preferred Reporting Items for Systematic Reviews and Meta-analysis Protocol.^[26]^ If there are any adjustments throughout the study, we will fix and update the details in the final report.

### 2.2 Eligibility criteria

#### 2.2.1 Study design

This study only included randomized controlled trials of vitamin D supplementation for type 2 diabetic microangiopathy. We will exclude observational, cohort, case-control, case series, and laboratory studies.

#### 2.2.2 Participants

The patients met the diagnostic criteria of T2DM proposed by the American Diabetes Association in 2010, regardless of race, gender and age. Diabetic microangiopathy of different degree.

#### 2.2.3 Interventions

This meta-analysis will include the randomized controlled trials of vitamin D supplementation regardless of dose and frequency. Trials will be included at least 4 weeks of treatment.

#### 2.2.4 Outcomes

The primary outcomes include patient before and after treatment: markedly effective: symptoms improved significantly > 70%; effective: symptoms reduced by 30% to 70%; ineffective: symptom improvement is < 30% or no improvement, or even worse. The nerve conduction velocity includes the sensory nerve conduction velocity and the motor nerve conduction velocity, which are evaluated by electromyography. In addition, urinary microalbumin/creatinine and fundus photography are also included. Secondary outcomes are mainly composed of fasting blood glucose and glycosylated hemoglobin, glomerular filtration rate, creatinine, uric acid, and adverse events.

### 2.3 Study search

We will search each database from the built-in until March 2020. The English literature mainly searches Cochrane Library, PubMed, EMBASE, and Web of Science, while the Chinese literature comes from CNKI, CBM, VIP, and Wangfang database. Simultaneously we will retrieval clinical registration tests and grey literatures. According to the PICOS principle, the keywords of our search terms were: (“vitamin D” OR “cholecalciferol” OR “25-hydroxyvitamin D2” OR “24, 25-dihydroxy vitamin D3”) AND (“Diabetic microangiopathy” OR “Diabetic Microangiopathy” OR “Microangiopathy, Diabetic” OR “Microangiopathies, Diabetic” OR “Diabetic nephropathy” OR “Diabetic Nephropathy” OR “Nephropathy, Diabetic” OR “Nephropathies, Diabetic” OR “Diabetic peripheral neuropathy” OR “Diabetic Peripheral Neuropathy” OR “Peripheral Neuropathies, Diabetic” OR “Diabetic retinopathy” OR “Diabetic Retinopathies” OR “Retinopathies, Diabetic”).

### 2.4 Study selection

**Figure 1:**
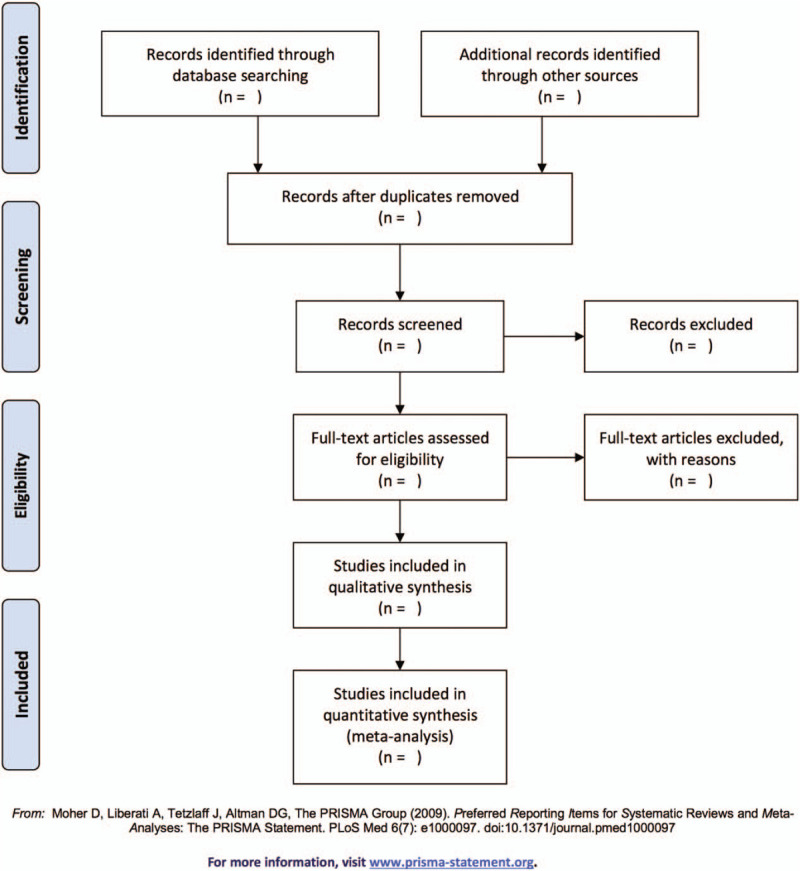
Flow diagram of study selection.

Two reviewers will retrieve all the literature independently. We will manage the electronic citations downloaded from these databases, which are located at endnote X8 for Mac (Thomson Reuters). The reviewers first screen the title and abstract of each citation to identify potentially eligible studies and then review the full text to confirm inclusion. negotiation with a third reviewer. A flow chart will be drawn to show the process of study selection (Fig. 1).

### 2.5 Data extraction and management

According to the characteristics of the study, we prepare an excel form for data collection before data extraction. Outcome indicators for eligible studies were independently extracted and filled in the data extraction form by 2 reviewers. If there is any argument, it can get an agreement by discussing through 2 reviewers or seek a third party's suggestion. The main data extracted are as follows: The first authors of the article, year of publication, fund source, interventions in experimental group, interventions in control group, time of treatment, course of disease, number of patients in each group, ages and sex of patients, outcomes and safety data. If you find something unclear in the study, you can contact the author of the communication directly for more detailed information. The above information was finally cross-checked by 2 reviewers.

### 2.6 Risk of bias assessment

All the included studies will be evaluated in accordance with the guidelines of Cochrane Handbook for Systematic Reviews of Interventions. Two review authors will independently evaluate the design.Bias risk through 7 assessment trials: random sequence generation (selection bias), allocation concealment (selection bias), blinding of participants and personnel (performance bias), blinding of outcome assessment (detection bias), incomplete outcome data (attrition bias), selective reporting (reporting bias), other bias. Each item is classified as “Low risk”, “High risk” or “Unclear risk”.^[27]^ The disagreement of bias risk will be resolved through further discussion or consultation to a third independent reviewer.

### 2.7 Statistical analysis

The risk ratio for dichotomous data will be calculated,respectively, along with 95% confidence interval (CI). For continuous data, the mean difference (MD) or standardized MD (SMD) with 95% CI will be estimated. If we use the same scale to measure an outcome in different studies, we will use MD. Similarly, if we use different scales to measure the same outcome, we will use SMD. If an outcome measure contains less than 2 trials, we will summarize the results descriptively.

Statistical heterogeneity among studies will be evaluated using the Cochran *Q* test (x^2^) and the *I*^2^ statistical value. We will categorize the heterogeneity using the following rules. *I*^2^ of 0% to 25% indicates low heterogeneity. *I*^2^ of 25% to 50% represents moderate heterogeneity. And *I*^2^ of 75% to 100% represents high heterogeneity. When the *P* value from a x^2^ test is more than .10 or *I*^2^ 50%, we will adopt the fixed-effects model. Otherwise, there will be perceptible differences between the studies. Subgroup analysis will be performed to identify possible explanations for statistical heterogeneity, taking into account prespecified factors.

We will utilize the Review Manage software V5.3.0 (The Nordic Cochrane Center, The Cochrane Collaboration, 2014, Copenhagen, Denmark) to statistically analyze all data. The overall risk ratio with its 95% CI for dichotomous data will be estimated. The MD or SMD with 95% CI will be calculated for continuous data in different situations. The fixed-effects model will be employed as appropriate for analysis. If the heterogeneity in the study is significant, subgroup analysis will be conducted to investigate possible sources of statistical heterogeneity. When a meta-analysis is not available, descriptive summaries of individual studies will be provided.

### 2.8 Additional analysis

#### 2.8.1 Subgroup analysis

If the results of the study are heterogeneous, we will conduct a subgroup analysis for different reasons. Heterogeneity is manifested in the following several aspects, such as race, age, sex, different intervention forms, drug dosage, treatment course.

#### 2.8.2 Sensitivity analysis

In order to investigate the stability of the results, we will conduct a sensitivity analysis for the outcomes. We will exclude each study that is included in the analysis 1 by 1, and then re-analyze and pooled the data and compare the difference between the reobtained effects and the original effects. In this way, we will be able to assess the impact of individual studies on the overall results and whether the results are reliable.

#### 2.8.3 Reporting bias

If there are >10 studies in the metaanalysis, the symmetry of the funnel plot will be assessed to examine publication bias, with results being interpreted cautiously. Grading the quality of evidence. In this systematic review, the quality of evidence for the entire study is assessed using the “Grades of Recommendations Assessment, Development and Evaluation (GRADE)” standard established by the World Health Organization and international organizations.^[28]^ To achieve transparency and simplification, the GRADE system divides the quality of evidence into 4 levels: high, medium, low,and very low. The GRADE profiler 3.2 will be employed for analysis.

## 3 Discussion

A meta-analysis of high-quality trials will provide the most reliable evidence for the clinical treatment of type 2 diabetic microangiopathy. The purpose of this systemic review and meta-analysis is to evaluate the effectiveness and safety of vitamin D supplementation in type 2 diabetic microangiopathy humans. We will identify the influence of effectiveness and safety in different dosages of vitamin D and different duration of treatment. Overall, we will give a comprehensive picture of efficacy and adverse events in patients treated with the vitamin D. In order to guarantee the accuracy and reliability of the results, the articles will be independently screened by different authors at least three times. Herein, this systemic review and meta-analysis will be the first to assess the effectiveness and safety of vitamin D supplementation in type 2 diabetic microangiopathy, which may offer a comprehensive understanding of vitamin D supplementation in type 2 diabetic microangiopathy.

### 3.1 Ethics and dissemination

In consideration of the systematic review of this protocol, ethical ratification is not required. In this study, participants were not recruited and data were not collected from participants. The review will be disseminated through peer-reviewed publications.

## Author contributions

**Conceptualization**: Junmin Chen, Xiayu Gong, Qiu Chen

**Data curation**: Jie Liu, Tingting Wang

**Funding acquisition**: Qiu Chen

**Investigation**: Xiaoyan Shi, Xiaoran Zhang

**Methodology**: Junmin Chen, Xiayu Gong, Qiu Chen

**Project administration**: Qiu Chen

**Software**: Junmin Chen, Xiayu Gong

**Supervision**: Xiaoyan Shi

**Validation**: Junmin Chen

**Writing – original draft**: Junmin Chen, Xiayu Gong

**Writing – review & editing**: Qiu Chen
